# **Clinical and patient-reported outcomes of distal femur fracture fixation in adults aged 18**–**50 years**

**DOI:** 10.1007/s00590-025-04392-4

**Published:** 2025-06-27

**Authors:** Wei Shao Tung, Robert K. Wagner, Jacob S. Borgida, Niels Brinkman, Maaz Muhammad, Adam N. Musick, Austin T. Gregg, Thomas J. Policicchio, Derek S. Stenquist, Thuan V. Ly

**Affiliations:** 1 Department of Orthopaedic Surgery, Massachusetts General Hospital, Boston, United States; 2 Harvard Medical School Orthopaedic Trauma Initiative, Boston, United States; 3 Department of Orthopedic Surgery and Sports Medicine, Amsterdam University Medical Centers, Amsterdam, Netherlands; 4 The University of Texas at Austin, Austin, United States

**Keywords:** Distal femur fracture, Young patients, PROMIS, Physical function, Nonunion, Infection

## Abstract

**Purpose:**

To describe nonunion and fracture-related infection (FRI) rates and patient-reported outcomes following operative treatment for distal femur fractures in young patients.

**Methods:**

We retrospectively identified all patients (aged 18–50) who were operatively treated for a distal femur fracture between 2006 and 2023 with ≥ 3-month clinical follow-up at two Level 1 Trauma Centers. Outcomes included reoperation for nonunion and FRI; and PROMIS physical function (PF), depression, and anxiety (reference population mean: 50). Multiple linear regression was performed to identify factors associated with PROMIS-PF.

**Results:**

Eighty-six patients met inclusion criteria. The median age was 34 years, 71% were male, 42% had an open fracture, and for 38 patients PROMIS scores were collected at an average of 9.8 years after treatment. Eleven patients (13%) required reoperation for nonunion and 3 (3.5%) for FRI. Median PROMIS-PF was 47.0 (IQR: 41.2–52.4), depression 45.8 (IQR: 38.9–53.6), and anxiety 46.7 (IQR: 39.5–60.5). PROMIS-PF was lower than the reference population score (*p* < 0.05). Increased age (1-year; ß: − 0.39; 95%CI: − 0.62 to − 0.17; *p* < 0.001) and BMI (1-unit; ß: − 0.59; 95%CI: − 0.98 to − 0.20; *p* = 0.004) at time of injury were associated with worse PROMIS-PF scores and longer follow-up (1-year; ß: 0.79; 95% CI: 0.27 to 1.3; *p* = 0.004) with better scores.

**Conclusion:**

One in 8 young patients with a distal femur fracture underwent reoperation for nonunion and one in 33 for FRI. Physical function scores were marginally lower than the reference population, whereas depression and anxiety scores were similar. The finding that physical function scores were more influenced by baseline patient factors than injury characteristics is important for prognostication and patient education. These results should be interpreted in the context of the small sample size, and future research with larger cohorts is needed to confirm these findings and better understand long-term functional outcomes in young patients following treatment of distal femur fractures.

**Supplementary Information:**

The online version contains supplementary material available at 10.1007/s00590-025-04392-4.

## Introduction

Distal femur fractures are severe injuries that occur in a bimodal distribution 1. In young patients, distal femur fractures are often the result of high-energy injury mechanisms, whereas low-energy falls are the primary mode of injury in elderly patients 2, 3. High-energy injury mechanisms may lead to polytrauma and a greater injury burden including open fractures, intraarticular fractures, and vascular injury. These characteristics have been associated with the development of nonunion [4–6. However, young patients often have more favorable comorbidity profiles, which may in turn improve functional recovery.

There has been a significant effort to improve outcomes following distal femur fractures. However, prior studies primarily included patients aged 50 years or older [4–8. Data from these studies may therefore not adequately characterize outcomes of younger patients.

The primary objective of this study was to describe (1) reoperation for nonunion and fracture-related infection (FRI) rates; and (2) long-term patient-reported outcomes measurement information system (PROMIS) physical function (PF), PROMIS depression, and PROMIS anxiety, and pain scores of young patients (aged 18–50 years old at time of treatment) who underwent operative treatment for distal femur fractures. The secondary objective was to compare PROMIS scores with normative reference population values and to identify patient and injury factors that were associated with PROMIS-PF scores.

## Methods

### Patients

After local Institutional Review Board approval (protocol number: 2023P003507), all consecutive patients (aged 18 to 50 years old at time of treatment) who underwent open reduction and internal fixation (ORIF) for a distal femur fracture (AO/OTA 33A or 33C) 9 between January 2006 and June 2023 at two level 1 trauma centers were retrospectively identified from an institutional database using Common Procedural Terminology codes 27511 and 27513. Exclusion criteria included (1) pathologic fractures, (2) patients with less than 3 months clinical and radiological follow-up (unless patient had healed and was discharged before 3 months follow-up), and (3) patients whose first surgery at the institution was a revision surgery. All eligible patients were contacted by telephone to collect PROMIS-PF CAT v2.0 10, PROMIS Depression CAT v1.0 11, PROMIS Anxiety CAT v1.0 11, and Numeric Pain Rating Scale (NPRS) scores. Patients were contacted 4 times before being considered lost to follow-up.

### Data collection

Explanatory variables were extracted from electronic medical records and included patient characteristics (age, body mass index [BMI], sex, tobacco use, diabetes mellitus, American Society of Anesthesiologists [ASA] physical status), injury characteristics (energy mechanism, AO/OTA fracture classification 9, Gustilo-Anderson classification 12, vascular injury, number and location of additional fractures, acute non-orthopedic surgical interventions, and Intensive Care Unit [ICU] stay), and treatment characteristics (staged treatment with cement spacer for acute bone loss, use of external fixation, and definitive fixation construct). Explanatory variables are displayed in Tables 1 and 2.

**Table 1:** Baseline patient and injury characteristics

Patient characteristics	N = 86^a^
Age, median (IQR)	34 (28–45)
BMI, median (IQR)	27 (23–30)
Male	61 (71)
Tobacco	30 (35)
Diabetes Mellitus	3 (3.5)
ASA	
I	19 (22)
II	43 (50)
III	24 (28)

*Injury characteristics*	
High energy mechanism	75 (87)
Penetrating injury	3 (3.5)
Number of additional upper extremity long-bone fracture(s)	
0	71 (83)
1	10 (12)
≥ 2	5 (5.8)
Number of additional lower extremity long-bone fractures(s)	
0	63 (73)
1	14 (16)
≥ 2	9 (10)
Additional pelvis fracture	9 (10)
Bilateral distal femur fractures	3 (3.5)
Acute neurosurgery intervention	4 (13)
Acute vascular surgery intervention	10 (12)
Acute general surgery intervention	7 (8.1)
Intensive Care Unit admission	26 (30)
Days in Intensive Care Unit, median (IQR)	5 (3–6)

^a^ Median (IQR), n (%); BMI, Body Mass Index; ASA, American Society of Anesthesiologists Physical Status; Missing values for: BMI (n = 6), tobacco use (n = 5)

**Table 2:** Baseline fracture and treatment characteristics

Fracture characteristics	N = 89^a^
AO/OTA classification	
A2	8 (9.0)
A3	11 (12)
C1	2 (2.2)
C2	28 (31)
C3	39 (44)
A2 (above knee arthroplasty)	1 (1.1)
Gustilo Type	
Closed	52 (58)
I	2 (2.2)
II	6 (6.7)
III	28 (31)
Open type unknown	1 (1.1)
Associated vascular injury	8 (9.0)

*Treatment characteristics*	
Staged treatment with cement spacer	4 (4.5)
Temporary external fixation	24 (27)
Days of external fixation, median (IQR)	4.0 (2.0—7.0)
Definitive fixation construct	
Single lateral locked plate	80 (90)
Intramedullary nail	4 (4.5)
Double plate	2 (2.2)
Blade plate	1 (1.1)
External fixation	1 (1.1)
Medial plate	1 (1.1)

^a^Median (IQR), n (%)

### Outcomes

The primary outcomes were reoperation for nonunion and FRI, level of physical function using PROMIS-PF, symptoms of depression using PROMIS depression, symptoms of anxiety using PROMIS anxiety, and current level of pain using the NPRS. Symptoms of depression and anxiety were also considered as explanatory variables to assess the association with level of physical function.

Nonunion was defined as any fracture requiring reoperation to promote union with staged revision of fixation or bone grafting as indicated by the treating surgeon. Bone grafting procedures following treatment with an antibiotic cement spacer in the setting of staged treatment for open fractures or fractures with bone loss were not considered nonunion procedures. FRI was defined as reoperation for infection meeting FRI consensus criteria 13. For all PROMIS measures, higher scores indicated higher levels of the measured outcome (e.g., higher PROMIS-PF scores indicate better physical function and higher PROMIS anxiety scores indicate higher levels of anxiety). A score of 50 represents the mean of the USA (US) reference population and every 10 points above or below represents one standard deviation (e.g., a score of 40 is one standard deviation lower than the mean of the reference population) 14. The NPRS, is an 11-point scale, with 0 representing no pain and 10 the worst imaginable pain.

### Statistical analysis

Descriptive statistics were summarized as medians with interquartile range (IQR) for all continuous variables and frequencies with percentages for categorical variables. Rates of nonunion and FRI were only analyzed descriptively. Differences in patient and injury characteristics between patients who reported patient-reported outcomes (PROs) and those without were assessed using Fisher’s exact test and Wilcoxon rank-sum test. The difference between PROMIS-PF and the US reference population mean of 50 was assessed using one-sample t-test as visual inspection of histograms demonstrated an approximately normal distribution of physical function scores, whereas differences between PROMIS anxiety and PROMIS depression and the US reference population mean were assessed using one-sample Wilcoxon Rank-sum test as data were not normally distributed. To identify patient and injury characteristics that were independently associated with PROMIS-PF at 9.8-year follow-up, a stepwise approach was used. First, univariate linear regression was performed to assess the association of explanatory variables of interest with PROMIS-PF. Next, variables with *p*-value < 0.10 in univariate linear regression and time from injury to PRO follow-up were included in a multivariable linear regression model. Symptoms of depression and symptoms of anxiety were highly correlated with a Spearman’s rank correlation coefficient of 0.86, and to avoid collinearity, symptoms of anxiety were included in the final model as this demonstrated the best model fit based on the R-squared statistic. The initial multivariable model included six variables. It is generally accepted that approximately 10 patients per independent variable are required in multivariable linear regression models, which would allow for four variables to be included in the model. Therefore, a stepwise elimination approach was used by removing the variable with the highest p-value until only variables with a *p*-value < 0.10 remained in the multivariable linear regression model. In multivariable linear regression analysis, patients with bilateral distal femur fractures had one fracture chosen at random as to not violate the statistical rule of independence. For one patient, missing BMI was replaced by median imputation. Statistical significance was set at a two-tailed a = 0.05. All data analyses were performed in R version 4.3.1 (The R Foundation for Statistical Computing, Vienna, Austria).

## Results

### Patient and injury characteristics

During the study period, 887 patients with AO/OTA type 33A or 33C femur fractures were treated operatively at one of the two institutions. After exclusion of pathologic fractures (*n* = 62) and patients with less than 3 months of orthopedic clinical or radiographic follow-up (*n* = 196), there were 629 patients remaining. Of these, 86 (14%) patients who underwent ORIF and were between the ages of 18 and 50 at the time of treatment were included. The median age at injury was 34 years (IQR: 28–45), 61 (71%) patients were male, and 30 (35%) patients were active smokers **(**Table 1**).** The majority of patients (*n* = 75, 87%) sustained a high-energy injury mechanism.

Three patients (3.5%) had bilateral distal femur fractures, resulting in a total of 89 distal femur fractures analyzed **(**Table 2**).** According to the AO/OTA classification, there were 26 (29%) Type A and 67 (75%) Type C fractures, with 37 (42%) open fractures, and 8 (9.0%) fractures associated with a vascular injury. Four (4.5%) fractures were treated in a staged fashion with an antibiotic cement spacer for a segmental defect, and 24 (27.0%) fractures were temporized with external fixation before definitive fixation. The majority of fractures were treated with a single lateral locking plate (*n* = 80, 90%).

### Reoperation for nonunion and fracture-related infection

Eleven patients (13%) required reoperation for nonunion and 3 (3.5%) for FRI **(**Table 3**).** One patient underwent a quadricepsplasty and extensor mechanism repair for arthrofibrosis with a quadriceps contracture, and ultimately underwent an above-knee amputation for persistent stiffness and poor function. One patient underwent a revision ORIF for nonunion and later underwent conversion to a distal femoral replacement for persistent nonunion.

**Table 3:** Outcomes

Surgical outcomes^b^	N = 86^a^
Reoperation for nonunion	11 (13%)
Reoperation for FRI	3 (3.5%)
Reoperation for symptomatic hardware	10 (12%)
Reoperation for arthrofibrosis and/or contracture	6 (7.0%)
Conversion DFR	1 (1.2%)
Amputation	1 (1.2%)
Patient-reported oucomes	n = 38^a^
PRO Follow-up (years)	9.8 (7.5–13.5)
PROMIS Physical Function	47.0 (41.2–52.4)
PROMIS Depression^c^	45.8 (38.9–53.6)
PROMIS Anxiety^c^	46.7 (39.5–60.5)
NRS pain	3.50 (2.00–5.75)

^a^ n (%), median (IQR); ^b^Numbers are not mutually exclusive (i.e., a single patient may have had reoperation for more than one indication); ^c^Outcomes missing for one patient; FRI, fracture-related infection; DFR, Distal Femoral Replacement; PRO, Patient-Reported Outcome; NRS, Numeric Pain Rating Scale

### Patient-reported outcomes

Seventy-nine of 86 (91.9%) eligible patients were available for contact (four patients were deceased, and three did not have contact information available). Thirty-eight of 79 (48%) responded at a median follow-up of 9.8 years (IQR: 7.5–13) after the injury. Patients who reported PROMs had an ASA classification of II more often than those that did not report PROMs **(**Appendix 1**).**

The median PROMIS-PF score was 47.0 (IQR: 41.2–52.4), which was lower than the US reference population average of 50.0 (*p* = 0.029) **(**Table 3**).** The median PROMIS depression score was 45.8 (IQR: 38.9–53.6) and PROMIS anxiety score was 46.7 (IQR: 39.5–60.5), which were not different compared to the US reference population of 50 (*p* = 0.179 and *p* = 0.786, respectively). The median NPRS for pain was 3.50 (IQR: 2.00–5.75).

### Factors associated with PROMIS-PF scores

In univariate linear regression analysis, BMI (*p* < 0.001), symptoms of depression (*p* < 0.001), symptoms of anxiety (*p* < 0.001), duration of PRO follow-up (*p* = 0.017), and ASA III (vs. ASA I-II, *p* = 0.047) were associated with level of physical function (Table 4). In multivariable analysis, higher age (1-year increase; ß: − 0.39; 95%CI: [− 0.62 to − 0.17]; *p* < 0.001) and BMI (1-unit increase; ß: − 0.59; 95%CI: [− 0.98 to − 0.20]; *p* = 0.004) were associated with worse levels of physical function, whereas longer follow-up duration (1-year increase; ß: 0.79; 95%CI: [0.27 to 1.3]; *p* = 0.004) was associated with better levels of physical function (Table 4). PROMIS anxiety scores were not significantly associated with levels of physical function (1-point increase; ß: − 0.19; 95% CI: [− 0.38, − 0.01]; *p* = 0.056). The final model explained 58% of the variance in PROMIS-PF scores.

**Table 4:** Univariate analysis for PROMIS Physical Function

	Univariate regression			Multivariable regression		
	Beta	95% CI	*P*-value	Beta	95% CI	*P*-value
Age (1-year increase)^a^	− 0.31	− 0.62 to 0.01	0.061	− 0.39	− 0.62 to − 0.17	** < 0.001**
BMI (1-unit increase)^a^	− 0.81	− 1.2 to − 0.37	** < 0.001**	− 0.59	− 0.98 to − 0.20	**0.004**
Gender						
Female (reference)	–	–	–			
Male	0.12	− 6.5 to 6.7	0.971			
Tobacco use^a^	2.1	− 4.7 to 9.0	0.530			
ASA^a^						
I-II (reference)	–	–	–			
III	− 7.1	− 14 to − 0.11	**0.047**			
High energy mechanism	− 3.4	− 18 to 11	0.640			
AO/OTA type C fracture	2.2	− 5.4 to 9.8	0.557			
Open fracture	6.1	− 0.03 to 12	0.051			
Any additional upper extremity long-bone fracture	− 0.26	− 8.6 to 8.1	0.950			
Any additional pelvis or lower extremity long-bone fracture	− 3.3	− 10 to 3.8	0.354			
Reoperation for nonunion or FRI	− 1.2	− 9.5 to 7.2	0.781			
PROMIS Depression (1 point increase)	− 0.44	− 0.66 to − 0.22	** < 0.001**			
PROMIS Anxiety (1 point increase)	− 0.41	− 0.61 to − 0.22	** < 0.001**	− 0.19	− 0.38 to 0.01	0.056
PRO Follow-up (1 year increase)	0.84	0.16 to 1.5	**0.017**	0.79	0.27 to 1.3	**0.004**

^a^at time of injury; CI, Confidence Interval; BMI, Body Mass Index; ASA, American Society of Anesthesiologists Physical Status; FRI, Fracture-related Infection; PRO, Patient-Reported Outcome

## Discussion

In this study of 86 young patients (aged 18–50 years old at time of treatment) with 89 distal femur fractures, one in 8 patients required reoperation for nonunion, and one in 33 patients required reoperation for FRI. After a median of 9.8 years following treatment, patients reported slightly worse PROMIS-PF scores compared to the US population reference, however depression and anxiety scores where similar. Higher age and BMI at the time of injury were associated with mildly worse levels of physical function, and longer follow-up duration was associated with mildly better levels of physical function.

The rate of revision surgery for nonunion was 13%, which is at the higher end of previously reported rates. Lee et al. 4 reported a nonunion rate of 10.9% in 1,111 patients with distal femur fractures (mean age 57 years, 50% intraarticular fractures, 30% open fractures). Risk factors for nonunion included Type 3 open fractures, intraarticular fractures, fracture comminution, and higher BMI 4. Stockton et al*.*^5^ reported an overall nonunion rate of 6% among 615 patients with distal femur fractures (median age 61 years, 52% intraarticular fractures, 23% open fractures). They stratified patients into three groups depending on their risk for nonunion based on baseline patient and injury characteristics. Patients with a higher baseline risk for nonunion were more often female, younger, had a relatively higher proportion of intraarticular fractures and open fractures, and a higher degree of fracture comminution. Cone et al. 6 reported on a cohort of 438 patients with similar characteristics to the patients in the present study (mean age of 48 years, 75% intraarticular fractures, and 38% open fractures). They observed a 13.9% nonunion rate, although fractures requiring second stage bone grafting after placement of an antibiotic spacer for segmental bone defects were also considered nonunions. Risk factors for nonunion included segmental bone loss, open fractures, chronic anemia, and increasing BMI. Combined, the findings from the current and previous studies consistently show that nonunion remains a significant complication after distal femur fracture treatment, specifically for more severe injury patterns and patients who are poor hosts. Of note, the majority (90%) of patients in the current study underwent single lateral locked plating. Studies increasingly show favorable biomechanical characteristics and healing rates with the use of dual-plate or nail-plate combinations [16–18. However, the current sample size was too small to allow for meaningful comparisons between these groups.

The FRI rate of 3.5% in the current study was at the lower end of previously published rates, ranging from 3 to 7% 15, 19. Brodke et al. 19 assessed risk factors for FRI after operative treatment for distal femur fractures and found that patients with infection were on average 5 years younger. However, they did not include age in multivariable analyses, and therefore, the independent effect of age on infection remains unclear. They identified alcohol abuse, intraarticular fractures, vascular injury, and increased surgical duration as risk factors for infection, whereas the use of local antibiotics was protective in multivariable analysis. Ricci et al. 15 identified a 4% infection rate in closed, and 7% in open distal femur fractures. They did not identify independent risk factors for infection in multivariable regression analysis. A possible explanation for the relatively low infection rate in the current study may be that younger patients typically have less comorbidities (e.g., diabetes mellitus and systemic vascular disease) which is known to influence infection rates. 20

In this study, the median PROMIS-PF score was lower than the average population score in the US (47 vs. 50) at an average follow-up of 9.8 years after treatment. The 3 points may underestimation given that younger patients have higher PROMIS-PF scores compared to the overall population average 21. Multivariable linear regression analysis demonstrated that higher age and BMI at baseline were associated with mildly worse physical function. Specifically, a 1-year increase was associated with a 0.39-point reduction in PROMIS-PF scores, and a 1-unit increase in BMI with a 0.59-point reduction. Longer follow-up duration was associated with better PROMIS-PF scores, with every additional year of follow-up being associated with a 0.79-point increase in PROMIS-PF. Considering that one standard deviation on PROMIS scores corresponds to a 10-point difference, the overall effect of the association between these factors and PROMIS-PF seems to be moderate. Interestingly, none of the injury characteristics or primary clinical outcomes (nonunion or FRI) were associated with level of physical function on univariate regression analysis. For example, intraarticular (AO/OTA Type C) are generally considered worse injuries than extra-articular fractures. The absence of an association may be due to confounding effects from other fracture characteristics (e.g., the degree of comminution and soft-tissue damage) or the small sample size. Regarding the primary clinical outcomes, it is possible that following (successful) revision for either nonunion or FRI, patients are able to achieve similar levels of physical function as those healing following the primary surgery. However, this should be interpreted carefully given the limited sample size. Nevertheless, these findings suggests that the trajectory of physical function recovery may be more influenced by baseline patient factors than the injury characteristics (including AO/OTA classification, energy mechanism, open fracture, additional pelvis or lower extremity fracture, or subsequent complications requiring reoperation), which may inform prognostication and patient education.

In univariate regression analysis, symptoms of depression and anxiety at follow-up were associated with lower physical function scores at follow-up. Interestingly, multivariable analysis did not show an association between symptoms of anxiety and physical function. This is contrary to evidence pointing to a strong correlation between psychosocial factors and outcomes such as levels of pain intensity and physical function after orthopedic trauma 22, 23. This may due to the relatively small sample size of 38 patients which increased the likelihood of a Type II error (failing to detect an effect when one exists); specifically given that the p-value of 0.056 for anxiety was close to the threshold for statistical significance. As such, early recognition of physical limitations as well as feelings of distress and unhelpful thoughts regarding symptoms remains important to identify people who are struggling in their recovery and may benefit from additional interventions aimed to reorient those thoughts and feelings. 24, 25

This study has several limitations. First, patients were treated at two urban academic institutions, which should be considered when generalizing results to other populations. Second, only 38 out of 79 contactable patients reported PROs (48%) and ASA classification differed between responders and non-responders. Warwick et al*.* demonstrated that women were more likely to respond to follow-up surveys 26. Similarly, in the current study, the majority of patients treated were male although relatively more women reported PROs. Third, the sample size of 38 limited the complexity (i.e., number of independent variables) of multivariable linear regression models, which may therefore not fully capture the complex relationship between patient, injury, and treatment characteristics and outcomes. Additionally, pre-injury physical, psychologic, and sociodemographic variables were not included in the assessment. Obtaining such measures from retrospective chart review is likely not sufficiently accurate due to the absence of standardized assessment, which is an inherent limitation of retrospective study designs. Fourth, most patients in this study were treated with lateral locked plating, whereas there is a current trend toward the use of dual-plate fixation or nail-plate fixation, both in the literature and clinically. Lastly, PROMIS scores were obtained at varying follow-up durations, which was associated with PROMIS-PF scores in univariate analysis. Follow-up duration was included in the multivariable regression analysis to account for this confounder.

## Conclusions

One in 8 young patients with a distal femur fracture required reoperation for nonunion and one in 33 for FRI. Level of physical function was lower than the US reference population at an average of 9.8 years after treatment, whereas symptoms of depression and anxiety were similar to the US reference population. The absence of a significant association between symptoms of anxiety and level of physical function may be secondary to the limited sample size and warrants further investigation. The finding that physical function scores were more influenced by baseline patient factors (i.e., age and BMI) than injury characteristics is important for prognostication and patient education to set realistic expectations for long-term physical recovery. These results should be interpreted in the context of the relatively small sample size, and future research with larger cohorts is needed to confirm these findings and better understand long-term functional outcomes in young patients following treatment of distal femur fractures.

## Supplementary Information

Below is the link to the electronic supplementary material.

Supplementary file1 (DOCX 18 KB)

## Data Availability

No datasets were generated or analysed during the current study.
