# Supplementary material for: Clinical and patient-reported outcomes of distal femur fracture fixation in adults aged 18–50 years
Source: Eur J Orthop Surg Traumatol. 2025 Jun 27;35(1):284. doi: 10.1007/s00590-025-04392-4 (PMC12204896; doi:10.1007/s00590-025-04392-4)
Supplement: Supplementary file 1 — Supplementary file1 (DOCX 18 KB) [file 590_2025_4392_MOESM1_ESM.docx]

| **Patient characteristics** | **No PROMs (N = 48)^a^** | **PROMs (N = 38)^a^** | **p-value^b^** |
| --- | --- | --- | --- |
| Age, median (IQR) | 34 (28 - 43) | 34 (28 - 46) | 0.670 |
| BMI, median (IQR) | 25.9 (22.6 - 29.7) | 27.7 (23.1 - 30.9) | 0.586 |
| Male | 38 (79%) | 23 (61%) | 0.093 |
| Tobacco use | 16 (33%) | 14 (37%) | 0.937 |
| Diabetes Mellitus | 3 (6.3%) | 0 (0%) | 0.251 |
| ASA |  |  | 0.041 |
| I | 15 (31%) | 4 (11%) |  |
| II | 19 (40%) | 24 (63%) |  |
| III | 14 (29%) | 10 (26%) |  |
| **Injury characteristics** |  |  |  |
| High energy mechanism | 39 (81%) | 36 (95%) | 0.102 |
| Penetrating injury | 0 (0%) | 3 (7.9%) | 0.082 |
| AO/OTA Type C fracture^c^ | 37 (77%) | 29 (76%) | 0.933 |
| Open fracture^c^ | 18 (38%) | 18 (47%) | 0.357 |
| Number of additional upper extremity long-bone fracture(s) |  |  | 0.825 |
| 0 | 40 (83%) | 31 (82%) |  |
| 1 | 6 (13%) | 4 (11%) |  |
| ≥2 | 2 (4.2%) | 3 (7.9%) |  |
| Number of additional lower extremity long-bone fractures(s) |  |  | 0.556 |
| 0 | 37 (77%) | 26 (68%) |  |
| 1 | 6 (13%) | 8 (21%) |  |
| ≥2 | 5 (10%) | 4 (11%) |  |
| Additional pelvis fracture | 6 (13%) | 3 (7.9%) | 0.725 |
| Bilateral distal femur fractures | 2 (4.2%) | 1 (2.6%) | >0.999 |
| Acute neurosurgery intervention | 3 (18%) | 1 (7.7%) | 0.613 |
| Acute vascular surgery intervention | 5 (10%) | 5 (13%) | 0.744 |
| Acute general surgery intervention | 4 (8.3%) | 3 (7.9%) | >0.999 |
| Intensive Care Unit admission | 14 (29%) | 12 (32%) | 0.818 |
| Days in Intensive Care Unit, median (IQR) | 5 (3 - 8) | 4 (3 - 6) | 0.715 |
| **Appendix 1 - Differences in patient and injury characteristics between patients with available PROMs and those without**  ^a^n (%), median (IQR); ^b^Wilcoxon rank sum test, Fisher’s exact test; ^c^For 3 patients with bilateral fractures, if either fracture was open or classified as AO/OTA Type C, the patient was considered to have an open or AO/OTA Type C fracture, respectively.  Abbreviations: BMI = Body Mass Index, ASA = American Society of Anesthesiologists Physical Status. | | | |
